# The Progression of Treatment for Refractory Hypercholesterolemia: Focus on the Prospect of Gene Therapy

**DOI:** 10.3389/fgene.2022.911429

**Published:** 2022-06-09

**Authors:** Zhi-Fan Li, Na-Qiong Wu

**Affiliations:** Cardiometabolic Center, National Center for Cardiovascular Diseases, Fuwai Hospital, Chinese Academy of Medical Science, Peking Union Medical College, Beijing, China

**Keywords:** refractory hypercholesterolemia, lipid lowering strategies, gene therapy, familial hypercholesterolemia, LDL- cholesterol, delivery system, gene editing

## Abstract

Refractory hypercholesterolemia (RH), including homozygous familial hypercholesterolemia (HoFH) and compound heterozygous familial hypercholesterolemia, is characterized by high levels of low-density lipoprotein cholesterol (LDL-C) despite existing cholesterol-lowering methods at maximal tolerable doses. Patients with RH have early onset and higher risk of atherosclerotic cardiovascular disease (ASCVD) under insufficient treatment. Therefore, it is urgent to seek new therapies to maintain the blood lipids in refractory hyperlipidemia at normal levels. Currently, new cholesterol-lowering strategies are on the market, not only at the protein level [i.e., bempedoic acid (inhibiting ATP-citrate lyase), alirocumab and evolocumab (monoclonal antibodies against PCSK9), evinacumab (monoclonal antibody against ANGPTL3)] but also at the transcript level [i.e., mipomersen (antisense oligonucleotide inhibiting ApoB), inclisiran (siRNA targeting PCSK9)], providing more options for RH patients to achieve their lipid-lowering targets. More RNA-based therapies targeting RH-related genes have been designed for the treatment. However, for a proportion of patients, especially those with LDLR deficiency, the available treatments are still insufficient. More recently, emerging genome engineering based on CRISPR/Cas9 techniques, and advanced delivery technologies such as lentiviral vectors, adenoviral vectors, adeno-associated viral vectors, lipid nanoparticles, and exosomes are being rapidly developed and implemented as novel therapies for RH. Gene therapy targeting RH-related genes has been successfully conducted in cells, mice, and non-human primates with high efficacy in lipid lowering and good tolerability. Especially the new generation of genome editing technique, base editing, performed *in vivo* with ideal lipid-lowering effect and limited occurrence of unwanted results. Excitingly, a phase I/II clinical study of LDLR gene replacement has been recently completed in RH patients, likely to be employed in clinical practice in the future. Furthermore, new targets for cholesterol reduction such as REV-ERB, G protein-coupled receptor, Ubiquitin specific peptidase 20 are continually being developed. This narrative review updates recent advances in treatment for RH, summarizes related clinical trials and preclinical studies, especially on the prospect of gene therapy.

## Introduction

Patients with refractory hypercholesterolemia (RH) have significantly elevated low-density lipoprotein (LDL) cholesterol levels despite treatment with lipid-lowering therapies at maximum tolerated doses, associated with a very high risk for atherosclerotic cardiovascular disease (ASCVD) (Mach et al., 2020; Rosenson et al., 2020). From an etiological perspective, this review defines RH as containing homozygous familial hypercholesterolemia (HoFH) and compound heterozygous familial hypercholesterolemia (HeFH), belonging to the category of hereditary metabolic disorders. HoFH is manifested by identical mutations in two alleles, and compound HeFH is manifested by double allele mutations (heterozygous genotypes with two mutant alleles at the same locus of two homologous chromosomes), which are associated with different mean LDL-C levels depending on the types of mutations (Hartgers et al., 2018). Historically, the prevalence of HoFH in the general population was believed to be 1/1,000,000, but recent studies in larger population have found this may be an underestimate (Austin et al., 2004; Cuchel et al., 2014; Beheshti et al., 2020). Using molecular genetic testing, the prevalence of HoFH or compound HeFH was estimated to be 1/300,000 in the Netherlands, at least three times higher than previously assumed (Sjouke et al., 2015; Bell and Watts, 2016). The FH prevalence among population with severe hypercholesterolemia incidence of complex heterozygosis is 23-fold higher than general population (Beheshti et al., 2020), and the clinical phenotype is similar to HoFH. Our team have investigated prevalence of clinical FH according to Dutch Lipid Clinic Network criteria in different Chinese patients, and found the prevalence of definite and probable FH was 3.5% in 8,050 patients with coronary artery disease (CAD) (5.8% in premature CAD) (Li et al., 2017), 3.9% in 1,843 patients with myocardial infarction (MI) (7.1% in premature MI) (Li et al., 2016), 6.5% in 1,093 patients 35 years of age with a first MI (Li et al., 2018), and 0.47% in 13,002 patients with first-onset acute MI (Shi et al., 2020).

Existing cholesterol-lowering methods include statins and ezetimibe, which have poor efficacy in patients with refractory hypercholesterolemia either single-agent or in combination (Hartgers et al., 2015). Proprotein convertase subtilisin-kexin type 9 (PCSK9) and angiopoietin-like protein 3 (ANGPTL3) have emerged as key regulators of LDL-C levels, in which loss-of-function mutation are associated with lower blood lipid levels and lower odds for ASCVD (Brandts and Ray, 2021). In recent years, scientists have developed new cholesterol-lowering drugs targeting different targets at different stages of cholesterol synthesis, transport and metabolism, such as microsomal transfer protein inhibitors (MTP inhibitors) (D’Erasmo et al., 2017), liver selective thyroid hormone mimics (Sjouke et al., 2014), ATP citrate lyase inhibitors (Brandts and Ray, 2020), monoclonal antibodies (mAbs) targeting PCSK9/ANGPTL3, oligonucleotides that inhibit apolipoprotein (Apo) B/ANGPTL3, and small interfering RNA (siRNA) targeting PCSK9/ANGPTL3 (Akoumianakis et al., 2021; Rached and Santos, 2021). Recently marketed new drug PCSK9 mAbs (Alirocumab and Evolocumab) has been gradually promoted and applied in extremely high-risk patients because of its significant reduction ability of LDL-C and ASCVD event risk (Sabatine, 2019). New lipid-lowering drugs including Bempedoic acid (inhibiting ATP-citrate lyase), Inclisiran (siRNA targeting PCSK9), Evinacumab (monoclonal antibody against ANGPTL3) have recently received U.S. Food and Drug Administration (FDA) and/or European Medicines Agency (EMA) approval (Aguilar-Salinas et al., 2021).

However, in clinical practice, patients with genetic LDLR deficiency do not respond well to PCSK9 mAbs, and for some patients with refractory hypercholesterolemia, the reduction of LDL-C still cannot fully meet their requirements of lipid standards despite current methods. Early in 2013, our team reported an 8-year-old HoFH boy with an early onset acute MI, detected very high levels of PCSK9 protein and lipid profile, and used strengthened lipid-lowering measures at that time (rosuvastatin 20 mg and ezetimibe 10 mg daily) to treat. However, we didn’t see significant improvement of his serum lipid profile (Wu et al., 2013). And in 2019, we reported a 33-year-old HoFH lady with variants in LDLR exon12: c.1724T.C (p.L575P) (NM_000527) had poor response to alirocumab (Wu et al., 2019). Besides, some drugs such as Mipomersen (antisense oligonucleotide inhibiting ApoB) (Raal et al., 2010) and Lomitapide (MTP inhibitors) (D’Erasmo et al., 2017), both of which can promote liver triglycerides accumulation and increase transaminase, causing nonalcoholic fatty liver disease, have limited clinical application due to side effects (Li et al., 2014; Blom et al., 2016). Therefore, there is a need for continuous research into new cholesterol-lowering targets and to develop new lipid-lowering drugs to meet the clinical needs of these patients.

RNA-based therapies are hot spots in drug development, enabling the desired outcomes such as knockdown of target genes and induced expression of selected proteins, and have been designed for the treatment of RH and other gene-specific diseases (Chi et al., 2017; Gupta et al., 2021). Gene therapy is a promising platform that not only offers an alternative to current lipid-lowering therapies, but may well be the ultimate cure for genetic diseases such as HoFH. However, how to apply this technology more widely depends on the guarantee of its safety and effectiveness (Cao et al., 2021).

Here, we describe recent progression on basic research and treatment of refractory hypercholesterolemia, focusing on advances in gene therapy.

## New Cholesterol-Lowering Strategies Targeting Transcription

### Antisense Oligonucleotides

Antisense oligonucleotides (ASOs) can target RNA, induce protein knockdown or restoration by activating RNA degradation or modulate pre-mRNA splicing, thus slowing disease progression. There are 10 approved ASO therapies in Europe and the United States, and a broad pipeline in development (Kuijper et al., 2021).

Mipomersen is an antisense oligonucleotide that binds to ApoB mRNA and subsequently down-regulates ApoB expression and VLDL production via ribosomes. Given subcutaneously 200 mg once a week, mipomersen reduced LDL levels by 21% in HoFH patients and 28% in HeFH patients (Hartgers et al., 2015). Phase III RCT trial studies showed that the addition of mipomersen in the maximum tolerance standard lipid-lowering therapy for HoFH patients significantly reduced the levels of LDL, ApoB and lipoprotein (a) [Lp(a)], by 25%, 27% and 31%, respectively (Raal et al., 2010). The most common adverse events with mipomersen included transient injection-site reactions and influenza-like symptoms, as well as elevated ALT in most patients. In 2013, mipomersen was approved by FDA as adjunctive therapy for HoFH treatment. However, it failed to pass the re-examination of the European Committee for Medicinal Products for Human Use due to concerns about the long-term impacts and adverse effects on the liver. Furthermore, the higher incidence of adverse events and intensive monitoring requirement impacted life quality of patients, thus limiting its clinical use (Chambergo-Michilot et al., 2022).

Angiopoietin-like protein 3 (ANGPTL3) and ANGPTL4 are members of the angiopoietin-like family of secretory factors that target lipoprotein lipase (LPL) and regulate lipid metabolism, primarily expressed in the liver (Ruscica et al., 2020). Functional loss variation in its genes is associated with significantly low plasma LDL and triglyceride levels, and prevention of atherosclerotic cardiovascular disease (Musunuru et al., 2010). Preclinical studies showed the significant effects of ASO targeting hepatic ANGPTL-3 mRNA on lipid metabolism in various mouse models, resulting in significant reductions of blood triglycerides (35%–85%), LDL cholesterol (7%–64%), and triglycerides within LDL particles, very-low-density lipoprotein (VLDL), and intermediate-density lipoprotein (Graham et al., 2017). However, study in cynomolgus nonhuman primates illustrated that platelet decreases were a common side effect of ASOs and might translate to humans (Henry et al., 2017).

Recently, a randomized, double-blind, dose-escalation phase I clinical trial of IONIS ANGPTL3-LRx (a drug targeting hepatic Angptl3 mRNA) was completed in healthy volunteers with elevated TGs and patients with FH to evaluate its safety, tolerability, pharmacokinetics, and pharmacodynamics (NCT02709850). 44 participants were randomly assigned into experimental groups: receiving an antisense oligonucleotide targeting ANGPTL3 mRNA in a single dose (20, 40, or 80 mg) or multiple doses (10, 20, 40, or 60 mg once weekly for 6 weeks), or corresponding placebo-controlled groups (0.9% sterile saline). Significantly reductions in absolute levels of ANGPTL3 protein (46.6%–84.5%, *p* < 0.01 for all doses) and non-HDL cholesterol (10.0%–36.6%) showed in all multiple-dose groups, values increasing corresponding to dose-escalation. Triglycerides (63.1 ± 10.9, *p* = 0.01) and VLDL (60.0 ± 15.5, *p* < 0.01) reached maximum lowering in the dose of 20 mg, and LDL reduced greatest in 60 mg group (32.9 ± 10.4, *p* < 0.01). No serious side effects were observed (Graham et al., 2017). More recently, phase II clinical trial of IONIS ANGPTL3-LRx carried out in participants with HoFH and other types of dyslipidemia. However, the former was withdrawn due to lack of available patients meeting entry criteria (NCT03455777) (Table 1). Therefore, whether ASO targeting ANGPTL3 can be applied to RH requires more clinical evidence.

**TABLE 1:** Registered clinical trials of RNA-based therapy and gene therapy for RH (clinicaltrials.gov; search date: 2022/05/11).

Indication	Title	Study type/CT phase	Therapeutic agent	Route of administration	Identifier	(Estimated) study start date–completion date
FH	Phase I Study of *Ex Vivo* Liver-Directed Gene Therapy for Familial Hypercholesterolemia	Interventional (Phase I)	autologous hepatocytes	Retrovirus LDL iv	NCT00004809	June 1992–1995
HyperTG, FH	Safety, Tolerability, Pharmacokinetics, and Pharmacodynamics of IONIS ANGPTL3-LRx in Healthy Volunteers With Elevated Triglycerides and Participants With Familial Hypercholesterolemia	Interventional (Phase I)	IONIS ANGPTL3-LRx	subcutaneous	NCT02709850	30 November 2015–26 June 2017
HoFH	AAV8-mediated Low Density Lipoprotein Receptor (LDLR) Gene Replacement in Subjects With Homozygous Familial Hypercholesterolemia (HoFH)	Interventional (Phase I/II	AAV8-hLDLR	iv infusion	NCT02651675	March 2016–27 November 2020
HoFH	A Long-term Follow-up Study to Evaluate the Safety and Efficacy of RGX-501	Observational	No investigational product, participants have previous received RGX-501(human LDLR Gene Therapy)		NCT04080050	30 September 2019–29 September 2025
HeFH, Elevated Cholesterol	Trial to Evaluate the Effect of Inclisiran Treatment on Low Density Lipoprotein Cholesterol (LDL-C) in Subjects With Heterozygous Familial Hypercholesterolemia (HeFH) (ORION-9)	Interventional (Phase III)	Inclisiran	SC injections	NCT03397121	28 November 2017–17 September 2019
ASCVD, Elevated Cholesterol	Inclisiran for Participants With Atherosclerotic Cardiovascular Disease and Elevated Low-density Lipoprotein Cholesterol (ORION-10)	Interventional (Phase III)	Inclisiran	SC injections	NCT03399370	21 December 2017 - 17 September 2019
ASCVD, ASCVD-Risk equivalents, Elevated Cholesterol	Inclisiran for Subjects With ASCVD or ASCVD-Risk Equivalents and Elevated Low-density Lipoprotein Cholesterol (ORION-11)	Interventional (Phase III)	Inclisiran	SC injections	NCT03400800	1 November 2017–27 August 2019
HCL	A Non-interventional Implementation Study to Evaluate Treatment With Inclisiran (Leqvio^®^) and Other Lipid Lowering Treatments in a Real-world Setting	Observational	Inclisiran	SC injections	NCT05362903	28 January 2022–31 January 2025
Dyslipidemias, FH, HyperTG	A Phase 1 Single and Multiple Dose Study to Evaluate the Safety, Tolerability, Pharmacokinetics and Pharmacodynamic Effects of ARO-ANG3 in Adult Healthy Volunteers and in Dyslipidemic Patients	Interventional (Phase I)	ARO-ANG3	SC injections	NCT03747224	7 January 2019–17 May 2021
Mixed Dyslipidemia	A Double-blind, Placebo-controlled Phase 2b Study to Evaluate the Efficacy and Safety of ARO-ANG3 in Adults With Mixed Dyslipidemia	Interventional (Phase II)	ARO-ANG3	SC injections	NCT04832971	28 June 2021–30 April 2023
HoFH	Phase 2 Study to Evaluate the Safety and Efficacy of ARO-ANG3 in Subjects With Homozygous Familial Hypercholesterolemia (HOFH)	Interventional (Phase II)	ARO-ANG3	SC injections	NCT05217667	June 2022–October 2023
FH	Exosome-based Nanoplatform for Ldlr mRNA Delivery in Familial Hypercholesterolemia	Interventional (Phase I)	LDLR mRNA exosomes	Intravenous/peritoneal infusion	NCT05043181	December 2021–December 2026

FH, familial hypercholesterolemia; HyperTG, hypertriglyceridemia; HoFH, homozygous familial hypercholesterolemia; HeFH, heterozygous familial hypercholesterolemia; ASCVD, atherosclerotic cardiovascular disease; HCL, hypercholesterolemia; SC, subcutaneous.

Moreover, recent studies provide new insights into the cholesterol-lowering effect of other regulators targeting LPL such as apolipoprotein (Apo) C-III and ANGPTL4 (Akoumianakis et al., 2021; Singh et al., 2021; Bellosta et al., 2022; Tardif et al., 2022; Xu et al., 2022). Currently, ASOs against Apo-A, and Apo-CIII are under development (Bellosta et al., 2022; Xu et al., 2022). Because of their great advantages in pharmacokinetics such as high affinity for plasma proteins and rapid distribution throughout the body, ASOs are potential cardiometabolic therapeutic strategies for treating refractory hypercholesterolemia and reducing the risk of cardiovascular disease (Bellosta et al., 2022). Further trials are needed to verify the clinical effects and provide detailed indications of these agents.

### Small Interfering RNA

In the clinical phase of treating hypercholesterolemia, small interfering ribonucleic acid (siRNA) technology is another important gene silencing approach targeting mRNA to block transcription (Mohamed et al., 2021).

Inclisiran is a small interfering ribonucleic acid (siRNA) that acts on PCSK9. In the phase III LDL-C lowering studies, adults with heterozygous FH (Orion-9 trial, NCT03397121) received a maximum dose of statins with or without ezetimibe and inclisiran (300 mg) subcutaneously on day 1, day 90, day 270 and day 450. LDL-C levels were significantly lower than those in the placebo group (Raal et al., 2020a), and adverse and serious adverse events were similar in both groups. Patients with ASCVD (Orion-10, NCT03399370) and patients at equivalent risk for ASCVD or ASCVD (Orion-11, NCT03400800) had elevated LDL-C levels despite receiving the maximum tolerated dose of statin (>100 mg/dl), randomized to inclisiran or placebo, and found that LDL-C levels decreased by approximately 50% in the inclisiran subcutaneously injected every 6 months (Ray et al., 2020). Inclisiran (Leqvio^®^) was approved by EC in December 2020 for the treatment of hypercholesterolemia and mixed dyslipidemia in adults and was approved by FDA in 2021, becoming the only siRNA drug currently on market to lower LDL-C. Recently, a real-world prospective observational cohort study to evaluate inclisiran (Leqvio^®^) and other lipid lowering treatments is recruiting, expecting to provide more clinical evidence in this newly initiated siRNA (NCT05362903).

A novel siRNA targeting ANGPTL3 mRNA, named ARO-ANG3, was developed by Arrowhead Pharmaceuticals and recently completed phase I study (NCT03747224) in dyslipidemic patients and in adult healthy volunteers. Results of ARO-ANG3 in hypercholesterolemia patients on a stable lipid-lowing regimen showed ANGPTL3 levels dropped by 79–88% on average, and mean maximum reductions in LDL-C was up to 42% (Arrowhead Reports Interim Clinical Data on Cardiometabolic Candidates Aro-Apoc3 and Aro-Ang3, 2020). In healthy volunteers, the reduction in fasting lipid profile was similar to that reported in ANGPTL3 deficient function carriers. Besides, this study showed good safety and tolerability of ARO-ANG3 (Watts et al., 2019). In HeFH patients, interim findings demonstrated that, from baseline to week 16, LDL-C reductions increased by 23%, 30% and 37% with rising doses (100, 200 and 300 mg), respectively (Watts et al., 2020). Currently, phase II studies of ARO-ANG3 in adults with mixed dyslipidemia (NCT04832971) and in participants with HoFH (NCT05217667) are ongoing, expected to provide more safety and efficacy information (Table 1).

## Gene Therapy of Refractory Hypercholesterolemia

### Virus-Mediated Gene Therapy

Gene therapy may provide a promising approach to treat RH because targeting specific genetic loci can lead to precise results with minimal side effects. Early experiments demonstrated that viral vector-associated gene transfer can up-regulate LDLR expression and control hypercholesterolemia in animal models (Andreoletti et al., 2001), and Grossman et al. first conducted a clinical trial in five HoFH patients using retrovirus-mediated gene therapy (NCT00004809) (Table 1). This treatment first transduced recombinant retroviruses carrying healthy LDLR with autologous hepatocytes *ex vivo*, then infused into patients’ livers directly. After 4 months, the trial achieved only a small proportion of normal LDLR expression, and a slight decrease in plasma total cholesterol, LDL-C, and ApoB (6–20%, 6–25%, 10–21%, respectively) only in 3 of 5 patients (Grossman et al., 1995). The unsatisfactory result was likely due to low transfection efficiency of retrovirus, thus selecting appropriate viral vectors may improve it.

The use of lentivirus vectors has improved *ex vivo* transduction efficiency, achieving transduction in nearly 90% of cells (Oertel et al., 2003). Ou et al. delivered Ldlr gene using lentiviral vector into FH mice and observed significant decrease of LDL-C by 46%, and amelioration of lipid accumulation (Ou et al., 2016). Hytonen et al. develop different virus-mediated gene transfer system in LDLR deficient rabbit model and found that lentiviral vector-LDLR resulted long-term reduction in lipid profile instead of AAV2, AAV9-LDLR (Hytönen et al., 2019).

Besides retrovirus and lentiviral vectors, adenovirus (Ad) vectors are effective in gene delivery. Transfer of the helper-dependent Ad vector LDLR gene into LDLR^−/−^ mice has been shown to ameliorate lipid profile and produce long-term protection against atherosclerosis (Nomura et al., 2004; Leggiero et al., 2019). Ad are also used in somatic genome editing without adverse consequences reported from Musunuru’ lab (Wang et al., 2016; Chadwick et al., 2017). Splicing regulation of ApoB is another approach that can treat FH. ApoB posttranscriptional modification appears to be safe and effective in lowering cholesterol by interfering with VLDL assembly and LDL clearance (Khoo, 2015). The peg technique appears to be useful for additional anti-inflammatory effects, and this modification does not interfere with LDL reduction and atherosclerosis regression induced by helper-dependent Ad vectors (Leggiero et al., 2013).

Adeno-associated virus (AAV) vectors are safe platforms and the most commonly used viral vectors for gene therapy delivery nowadays (Xu et al., 2019). Different serotypes of AAV-based vectors have different organ-specific tropism. Among them, hepatotropic vectors based on AAV serotype 8 (AAV8) have been showed best in aspects of total cholesterol profile reduction and hepatocyte transduction (Lebherz et al., 2004), thus been developed for liver-directed gene therapy including FH.

AAV8-mediated gene therapy is widely used and suitable for somatic genome editing. Inducible degrader of LDLR (IDOL), an E3-ubiquitin ligase that binds LDLR at different locations with PCSK9 can promote receptor ubiquitination and lysosomal degradation, having a positive effect on LDL metabolism through specific amino acid substitution (Yu et al., 2021). A study of humanized mice showed that AAV8-mediated expression of IDOL in the liver resulted in increased LDL dependence on LDL plasma levels (Ibrahim et al., 2016). A recent toxicological study assessed the effect of AAV8 on direct expression of LDLR in rhesus monkeys, suggesting that the treatment is safe except for mild and transient transaminases and immune adaptation responses (Greig et al., 2017a). In addition, AAV8-induced RNA silencing against ApoB (via short hairpin RNA and artificial microRNA) has led to a significant reduction in plasma cholesterol (Maczuga et al., 2014).

Currently, FDA has already approved two therapies of AAV-mediated gene therapy. One is AAV2-mediated delivery of the RPE65 gene in treating confirmed biallelic RPE65-mediated inherited retinal dystrophy in 2017, the other is AAV9-mediated therapy that functionally replaces the mutated survival motor neuron 1 gene in treating spinal muscular atrophy type 1 disease in children under 2 years of age. Clinical trials have confirmed the significant efficiency and safety of these AAV-mediated therapies (Mendell et al., 2017; Russell et al., 2017; Pennesi et al., 2018). The most related clinical trial of RH treatment is AAV8-mediated delivery of LDLR gene for HoFH, which was completed as a phase I/II study in 2020, while no study results were posted yet (NCT02651675) (Table 1).

Before initiating of phase I/II clinical trial, Greig et al. (2017b) determined the pharmacology and toxicology of clinical candidate vector, AAV8.TBG.hLDLR, as well as those expressed mouse LDLR, AAV8.TBG.mLDLR in a specific mouse model of HoFH. They used 280 homozygous double Ldlr/Apobec-1 knockout mice which can develop severe hypercholesterolemia, and divided them into 5 cohorts. Four cohorts used AAV8.TBG.mLDLR in different doses (7.5 × 10^11^ GC/kg, 7.5 × 10^12^ GC/kg, and 6.0 × 10^13^ GC/kg) as vectors and the other used AAV8.TBG.hLDLR (6.0 × 10^13^ GC/kg). The lowest dose stood for the initial dose of the ongoing clinical trial (NCT02651675), the middle dose represented the highest clinical trial proposed dose, while the highest dose is approximately 8-fold the middle dose to test the safety. Pathology analysis indicated no dose-limiting toxicities even in the highest dose despite mild and transient liver pathology. Therefore, the maximally tolerated dose was higher than 6.0 × 10^13^ GC/kg while the no-effect dose was equal to or higher than the middle dose of 7.5 × 10^12^ GC/kg. They also determined the minimally effective dose was 7.5 × 10^11^ GC/kg, based on measurable reductions in total serum cholesterol, and suggested the therapeutic window for the treatment of HoFH was no less than 80-fold (Greig et al., 2017b). Those findings were considered being clinically significant and provided evidence for proposed clinical trials.

In non-human primate (NHP), PCSK9 knockdown by AAV-delivered meganuclease was demonstrated to be effective, safe and long-term durable (Wang et al., 2018; Wang et al., 2021). Wang et al. designed a combination of AAV8 and engineered meganucleases named M1PCSK9 (first generation) and M2PCSK9 (second generation) to target a sequence in PCSK9 gene exon 7 which was conserved between humans and macaques. After administering AAV8-M1PCSK9 into four macaques, all animals achieved dose-dependent PCSK9 inactivation in liver and serum within 6 weeks as well as stable serum cholesterol decrease up to 11 months. The modest dose (6.0 × 10^12^ GC/kg) of AAV8-M2PCSK9 injection into two other macaques led to a 34% reduction of serum LDL. Besides, AAV8-M2PCSK9 was more precise in target recognition and had less off-target cleavage (Wang et al., 2018). During the following 3-year monitoring, researchers found the all abovementioned six macaques, and another two treated with the AAV3B-M2PCSK9 vector maintained PCSK9 knockdown by AAV8-M2PCSK9 and serum LDL-C level reduction. Human genetics data indicated therapeutic potential of AAV meganuclease and chimeric liver-humanized mouse model showed similar on-target editing in primary human hepatocytes. No obvious adverse effects except transient transaminitis during early phase were detected, and most hepatocytes sustained stably in histopathology (Wang et al., 2021).

Nonetheless, the possibility of detrimental immune response to virus-mediated gene delivery hampered its use in genetic therapy. The first pilot study in which retrovirus genes were transferred to the liver cells of HoFH patients resulted in a different biochemical response (Grossman et al., 1995). Additionally, mild toxicity of AAVs is reported at high doses from animal studies, hence the need to establish more effective gene therapy approaches (Lau and Suh, 2017).

### Non-viral Vectors for Gene Therapy

In order to overcome the difficulties of host immune response of viral vectors, novel vectors with lower toxicity and immunogenicity are under development. Hou et al. demonstrated the creation of multiple minicircle non-viral DNA vectors. After specific modifications and highly efficient liver-specific LDLR gene expression, correction of hypercholesterolemia in LDLR deficient mice has no significant toxicity, thus providing another potential genetic tool for treating FH (Hou et al., 2016). In addition, human induced pluripotent stem cell (hiPSC) technology has shown encouraging results through plasmid vectors (Hou et al., 2016). Recently, a study expressed LDLR cDNA binding microRNA in FH mouse model with episomal non-viral vector, which inhibited the 3-hydroxy3-methylglutarate reductase (HMG-COA) and resulted in lipid level lowering about 32% in animals (Kerr et al., 2016).

Previous studies established that polymer nanoparticles designed for specific purpose are efficient in siRNA, microRNAs and drugs delivery (Maestro et al., 2021). Lipid nanoparticles (LNPs) are formed by amphiphilic lipids, suitable as vehicles for nucleic acid delivery. Yin et al. developed a LNP coencapsulated chemically enhanced single guide RNAs (sgRNA) and Cas9 mRNA to knockout PCSK9 gene in mouse liver, and detected a significant reduction of serum PCSK9 as well as a 35%–40% total cholesterol decrease (Yin et al., 2017). A recent study determined that PCSK9-targeted zinc finger nuclease mRNAs formulated into LNP could effectively delivered to the liver through intravenous injection in mouse models, achieving more than 90% knockout of the PCSK9 gene expression after the first dose. Besides, this method was well tolerated and efficient genome editing continuously increased after repeated dosing (Conway et al., 2019).

Compared with nanoparticles constructed from artificial materials, endogenous nano-sized carriers have the distinct advantage of good *in vivo* biocompatibility, avoiding rapid recognition and clearance by the reticuloendothelial system (Baek et al., 2019). Exosome (diameter of 30–150 nm) is a cell-derived vesicle released by most eukaryotic cells, which is an important medium of cell communication and material transmission by carrying proteins, non-coding RNAs, DNA, and other bioactive substances (Yang et al., 2020). Based on their endogenous, biocompatible and multifunctional properties, exosomes are emerging as a new tool for drug delivery systems and precision therapy. Recently, an exosome-based Ldlr gene therapy for FH successfully implemented in mice. Li et al. generated exosomes encapsulating enriched Ldlr mRNA by forced expression of Ldlr in AML12 cells. After confirming Exo^Ldlr^ could efficiently deliver functional Ldlr mRNA *in vitro*, investigators injected Exo^Ldlr^ in atherosclerotic Ldlr^−/−^ mice. Those exosomes successfully delivered wild-type Ldlr to the liver and expressed LDLR protein, significantly decreasing LDL-C, total cholesterol, and triglyceride levels, without any noticeable adverse effects. Moreover, Exo^Ldlr^ reduced lipid deposition in the liver and atherosclerotic lesions (Li et al., 2021). More recently, a first-in-human study of an exosome-based nanoplatform for Ldlr mRNA delivery is currently in phase I clinical trials (NCT05043181), promising to treat HoFH patients (Table 1).

**FIGURE 1:**
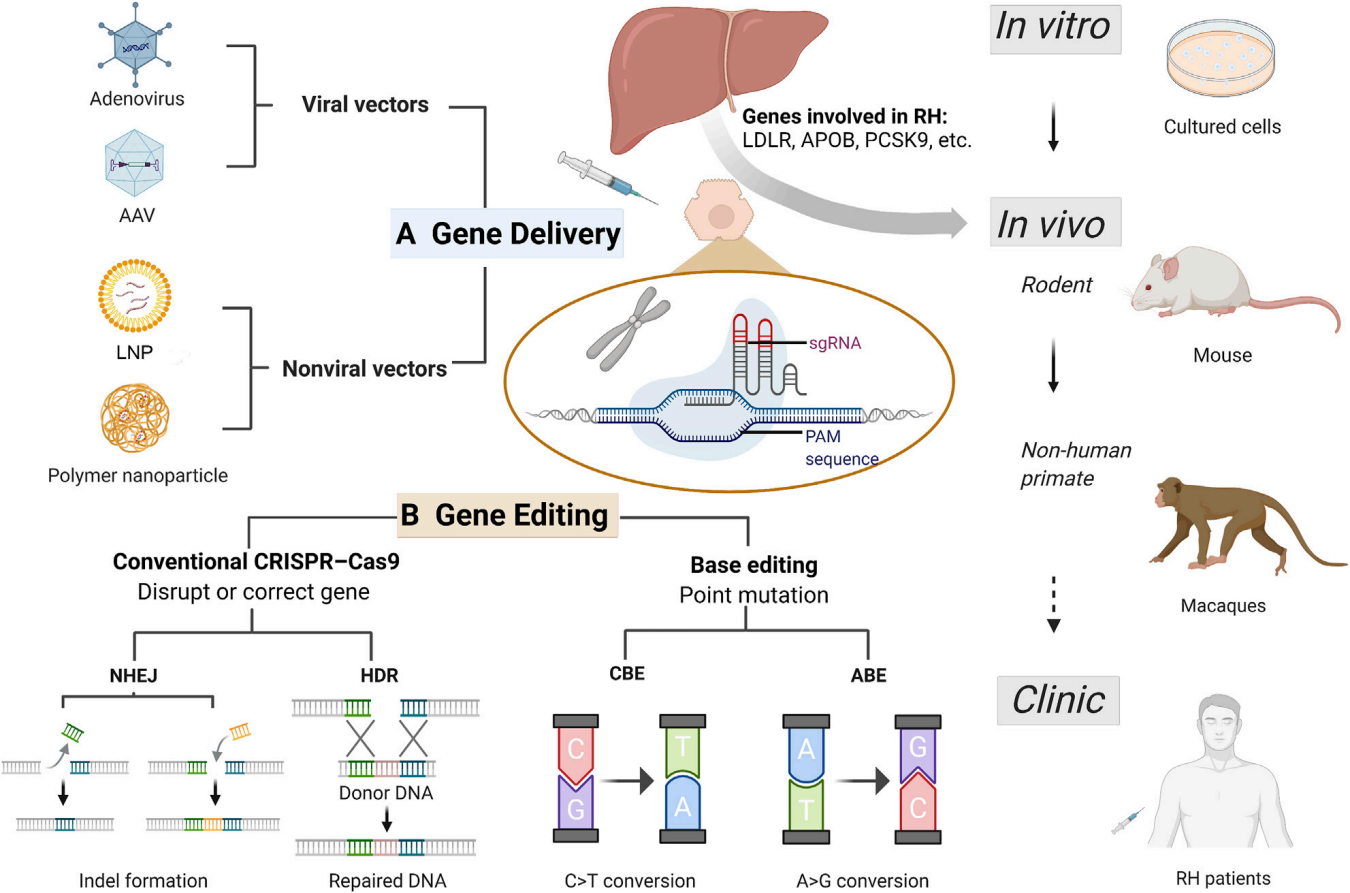
Current CRISPR/Cas9 based gene therapy for refractory hypercholesterolemia. Refractory hypercholesterolemia (RH) including homozygous familial hypercholesterolemia and compound heterozygous familial hypercholesterolemia, associated with mutations in genes encoding LDLR, APOB, PCSK9, etc. Efficient vectors enable delivery of CRISPR progenitors and genetic materials *in vitro* and *in vivo*, and the development of CRISPR technology has improved its efficacy and safety in target gene editing. Gene therapy experiments have been successfully conducted in cells (including human induced pluripotent stem cell), mice and non-human primates, expected to be used for clinical treatment in the future. **(A)** Common delivery vectors used in RH gene therapy. Adenovirus and adeno-associated virus are the most frequently used viral vectors in RH gene therapy. Non-viral vectors including lipid nanoparticle and engineered polymer nanoparticles offer more options for gene delivery with greater capacity and less toxicity and immunogenicity. **(B)** Gene editing based on CRISPR/Cas9 system for treating RH. For RH-related genes, gene disruption and correction can be achieved using conventional CRISPR-Cas9 and base editing techniques. In conventional CRISPR-Cas9, NHEJ can induce indel mutation (insertion or deletion), achieving disruption of pro-atherosclerotic genes such as PCSK9 and ANGPTL3; and HDR can correct a disease-causing mutation from donor DNA. Base editing is a new generation of CRISPR technology that enables single-nucleotide changes without double-strand breaks for precise gene editing. CRISPR-Cas9, clustering regularly spaced short palindromic repeats (CRISPR) and CRISPR-associated protein; RH, refractory hypercholesterolemia; AAV, adeno-associated virus; LNP, lipid nanoparticle; PAM, protospacer-adjacent motif; sgRNA, single guide RNA; NHEJ, non-homologous end joining; HDR, homology-directed repair; CBE, cytosine base-editors; ABE, adenine base-editors.

Common gene therapy delivery vectors suitable for both *in vitro* and *in vivo* experiments to treat RH were briefly summarized in Figure 1A. In conclusion, almost all delivery methods have both advantages and disadvantages. Further efforts are needed to quest for appropriate delivery systems possessing rare toxicity and immunogenicity, high transfection rate efficiency, high specificity to targets, large capacity, and ease of manipulation (Sharma et al., 2021).

### CRISPR/Cas9 Gene Editing

Clustering regularly spaced short palindromic repeats (CRISPR) and CRISPR-associated protein (Cas) system is currently the most widely used programmable genome editing tool.

CRISPR/Cas9 system can be oriented to recognize protospacer-adjacent motif (PAM) sequence at the targeted location by an artificial single guide RNA (sgRNA), and cleave DNA strands by the Cas9 nuclease (Liu et al., 2022). Then the double-strand DNA breaks can be repaired by the cell’s natural repair machinery, non-homologous end joining (NHEJ), to restore the original DNA sequence or introduce insertion and deletion (indel mutation); or by using another customized DNA repair template to realize homology-directed repair (HDR) (Porteus, 2019) (Figure 1B).

Currently, *streptococcus* pyogenes Cas9 has been widely used in genome engineering (Bolukbasi et al., 2016). Other *in vivo* delivery of Cas9 orthologs including *staphylococcus aureus* Cas9 (SaCas9), *campylobacter jejuni* and *neisseria meningitidis* Cas9 (NmeCas9) (Esvelt et al., 2013; Kim et al., 2017a). Ran et al. used a single AAV vector to deliver SaCas9 and its sgRNA to target mouse liver, and observed more than 40% Pcsk9 gene modification within 7 days after injection, along with substantial decreases in blood Pcsk9 and total cholesterol (Ran et al., 2015). Ibraheim et al. validated the efficiency of *in vivo* delivery of NmeCas9 guided by all-in-one recombinant AAV to target Pcsk9 gene in C57Bl/6 mice, and determined significant reduction in serum cholesterol levels (Ibraheim et al., 2018). Jiang et al. demonstrated a non-viral system, capable of *in vivo* delivering CRISPR/Cas9 components targeting of mouse Pcsk9 gene, and observed a high cutting efficiency in the targeted region, accompanied by a reduction in PCSK9 protein (Jiang et al., 2017).

CRISPR correction of LDLR dysfunction is a promising therapeutic strategy for RH treatment, especially for those HoFH patients bearing a null variant in LDLR gene. Transformed differentiated hepatocellular like cells (HLCs) showed increased LDL uptake and FH phenotypic modification, either by vector or specific genome editing by CRISPR/Cas9 techniques (Kuijper et al., 2021). Okada et al. (2019) studied the recovery of LDLR function and immunogenicity of gene-corrected iPSC-derived HLCs from an HoFH patient harbored a point mutation in exon 6 of LDLR using the CRISPR/Cas9 method. They reprogrammed T-cells of the HoFH patient to iPSCs and then differentiated into HLCs. Both homozygous gene-corrected iPSC-derived HLCs clone and two heterozygous clones successfully gained functional recovery of LDL uptake. Additionally, the immunogenicity after gene correction against the HoFH patient’s peripheral blood mononuclear cells was low and similar to that before operation. Therefore, LDL uptake of HoFH-iPSC can be repaired by CRISPR correction without further immunogenicity, suggesting this gene-corrected strategy has clinical potential in the treatment of HoFH (Okada et al., 2019).

A more recent preclinical study applied CRISPR/Cas9 system delivered by AAV to realize gene editing of Ldlr in mutant knock-in mouse model, and determined *in vivo* AAV-CRISPR/Cas9 somatic cell gene editing could correct LDLR mutations and ameliorate hypercholesterolemia. After confirmation of the efficient mouse model generated the E208X nonsense point mutation in the Ldlr gene, researchers delivered elaborate AAV-CRISPR/Cas9 into newborn Ldlr (E208X) mice to correct Ldlr gene mutations in a subset of hepatocytes. When mice matured, the AAV-CRISPR/Cas9 treatment group had approximately 6.7% (6.67 ± 0.64%) correction of the Ldlr alleles, and partially (∼18%) expression of LDLR proteins. Compared with same high-fat diet control group, the treated group had greater reductions in total cholesterol, triglycerides, and LDL-C. The symptoms of atherosclerosis were significantly reduced, as were the symptoms of macrophage infiltration and lipid accumulation. No obvious off-target or immune rejection reaction was found in adult mice, indicating the efficacy and safety of the gene editing therapy (Zhao et al., 2020).

The above-mentioned evidences point to CRISPR/Cas9 as a viable genome editing tool against RH, particularly in cases involving LDLR deficiency. However, there are several technical limitations and safety concerns of CRISPR/Cas9 which limit its application in clinical. First, because of DNA double-strand breaks introduced in CRISPR/Cas9 technology, unpredictable repair such as random insertions and deletions (indel) may occur and results in on-target mutagenesis. Additionally, low-rate HDR mediated editing leads to inefficient on-target alterations and is limited to dividing cell types. Besides, off-target mutagenesis due to guide RNA lack of sufficient specificity would increase the therapeutic risks, thus hinder research and clinical application (Fu et al., 2013; Doudna, 2020).

### Base Editing

Base editing is a new generation of genome editing techniques and can solve undesired effects of CRISPR/Cas9 (Anzalone et al., 2020). Base editor toolbox utilizes the positioning and cleavage capabilities of the Cas system for direct and precise single base substitution, without inducing double-stranded DNA breaks (Rees and Liu, 2018). Two classes of DNA base-editors have been described: cytosine base-editors (CBEs) and adenine base-editors (ABEs), realizing installation of all four transition mutations (C→T, T→C, A→G, and G→A) (Kantor et al., 2020) (Figure 1B).

Previous study demonstrated that the third-generation base editor (BE3), a widely used CBE which converts a targeted C G base pair to a T A base pair, resulted in fewer off-target mutagenesis than standard CRISPR/Cas9 *in vitro* (Kim et al., 2017b). Chadwick et al. established the efficacy of base editing *in vivo* for the first time by delivering base editor to the codon Trp-159 site of the mouse PCSK9 gene. After injecting BE3 with PCSK9-targeting gRNA in adult mice, researchers observed a median rate of 25% base editing in PCSK9 alleles, associated with more than 50% reduction in blood PCSK9 protein levels. Indel mutagenesis rate (approximately 1%) was far lower than that in prior studies of *in vivo* genome editing (∼40%) (Wang et al., 2016). Although the degree of plasma cholesterol decrease (∼30%) was less than that in standard CRISPR/Cas9 genome editing (35%–40%) (Ding et al., 2014), it is enough to reduce cardiovascular risk when translated to humans. Moreover, no evidence of off-target mutagenesis was obtained in this *in vivo* study.

Following PCSK9, *in vivo* base editing of ANGPTL3 was demonstrated to be successful. Chadwick et al. (Chadwick et al., 2018) identified the codon Gln-135 of ANGPTL3 gene was targetable for base editing. AAV encoding BE3 with and without gRNA targeting Angptl3 Gln-135 was injected in 5-week-old male C57BL/6J mice and BE3-Angptl3 caused a median editing rate of 35% increase in the Angptl3 target site, associated with significantly lower in mean levels of plasma ANGPTL3 protein (50%), triglycerides (35%), and cholesterol (20%) compared to BE3 without gRNA (BE3-control). Besides, BE3-Angptl3 performed better than BE3 targeting Pcsk9 in declining triglycerides. In male hyperlipidemic Ldlr-knockout mice (phenocopy of homozygous FH which PCSK9 loss-of-function has little effect) of the same age, BE3-Angptl3 showed higher efficiency (as compared to the BE3 control), with 56% reduction in triglycerides, and 51% reduction in cholesterol levels (Chadwick et al., 2018). Additionally, there was no evidence of off-target mutagenesis, and decrease of bone marrow hematopoietic stem cells which documented in ANGPTL3 full knock-out mouse (Zheng et al., 2011) was not observed in BE3-Angptl3-treated mice.

Recently, a study in humanized mouse model target human PCSK9 showed similar results. Researchers first generated a knock-in mouse model with liver-specific expression of human PCSK9 (hPCSK9-KI), and compared the lipid lowering efficiency of three treatment: evolocumab, CRISPR/Cas9-mediated genome editing, and base editing using gRNA. All treatments resulted in reduction of cholesterol levels in hPCSK9-KI, whereas base editing was the only one reducing levels of both human and mouse circulating PCSK9 protein (∼32% and ∼28%, respectively). Compared to Cas9-mediated genome editing, BE3 was more precise with no detectable off-target editing or chromosomal translocations *in vivo*, generated fewer indels, and potentially reduced immune response probability (Carreras et al., 2019).

Genome-wide profiling of ABE showed lower off-target rates compared to canonical SpCas9 (Liang et al., 2019). More recently, Musunuru et al. took a step toward investigating the efficacy of base editing in liver-specific PCSK9 of nonhuman primates. After a single-dose of LNP loaded with ABE injecting into cynomolgus monkeys, researchers observed almost complete editing of Pcsk9 in the liver. Circulating levels of PCSK9 and LDL-C were stably reduced by nearly 90% and up to 60%, and lasted for at least 8 months (Musunuru et al., 2021). Rothgangl et al. detected that ABEmax, a codon-optimized version, delivered by LNP to target liver provided that up to 67% knockdown of Pcsk9 in mice and 34% in macaques, resulting in concomitant reductions in plasma PCSK9 proteins and LDL levels of approximately 95% and about 58% in mice, and 32% and 14% in nonhuman primates, respectively (Rothgangl et al., 2021). Experimental studies of base editing technology for the treatment of RH were shown in Table 2.

**TABLE 2:** Experimental studies of base editing for RH.

Species	References	Target	Cas nuclease	Model	Delivery
Mouse	Chadwick et al. (2017)	Pcsk9 (Trp-159 site)	BE3	5-week-old male C57BL/6J mice	Adenoviral vector
	Carreras et al. (2019)	mouse Pcsk9 and human PCSK9	BE3	10-week-old male hPCSK9-KI mice and wildtype mice	Adenoviral vector
	Chadwick et al. (2018)	Angplt3 (Gln-135 site)	BE3	5-week-old male C57BL/6J mice	Adenoviral vector
		Angplt3+Pcsk9	BE3	5-week-old male C57BL/6J mice	Adenoviral vector
		Angplt3	BE3	5-week-old male B6.129S7-Ldlrtm1Her/J mice	Adenoviral vector
	Rothgangl et al. (2021)	PCSK9	ABEmax	mice	Lipid nanoparticle
Macaque	Musunuru et al. (2021)	PCSK9	ABE8.8-m	cynomolgus monkeys	Lipid nanoparticle
	Rothgangl et al. (2021)	PCSK9	ABEmax	cynomolgus macaques (Macaca fascicularis)	Lipid nanoparticle

Species	References	Editing rate	Decreased levels of target protein	Degree of plasma cholesterol decrease
Mouse	Chadwick et al. (2017)	Day 5: a median rate of 25% (average rate of 24%, max = 34%); Week 4: median rate of 28%	Day 5: PCSK9 protein (∼56%); Week 4: PCSK9 protein (∼54%)	Day 5: cholesterol (28%); Week 4: cholesterol (28%)
	Carreras et al. (2019)	most in the human PCSK9 and mouse Pcsk9 were the targeted C to T transitions	Week 3: human PCSK9 protein (∼32%); mouse PCSK9 protein (∼28%)	significant reductions in total cholesterol
	Chadwick et al. (2018)	Day 7: a median editing rate of 35%	Day 7: mean levels of plasma ANGPTL3 protein (49%)	Day 7: triglycerides (31%); cholesterol (19%)
		-	Day 7: ANGPTL3 protein (∼48%); PCSK9 protein (∼57%)	Day 7: triglycerides (∼27%); cholesterol (∼29%)
		-	Day 14: ANGPTL3 protein (∼24%)	Day 14: triglycerides (56%); cholesterol (51%)
	Rothgangl et al. (2021)	67%	∼95%	LDL-C 58%
Macaque	Musunuru et al. (2021)	almost complete editing of PCSK9	nearly 90%	LDL-C up to 60%
	Rothgangl et al. (2021)	34%	32%	LDL-C 14%

PCSK9, proprotein convertase subtilisin-kexin type 9; ANGPTL3, angiopoietin-like protein 3; BE3, third-generation base editor; ABE, adenine base-editor.

Base editing functions without inducing double-strand DNA breaks, limiting the occurrence of unwanted mutations and chromosomal abnormalities (Komor et al., 2018). Therefore, CRISPR base editing could be a promising strategy in correcting LDLR gene mutations or multiple genes defects and treating RH, and may help improve the efficacy of current lipid-lowering drugs.

### Concerns Beyond Technology of Gene Therapy

Although gene therapy is an emerging therapy with great potential to treat or cure serious diseases, potential risks of novel drugs should always be carefully considered, especially at the aspect of human genome editing.

In 2017, the US National Academies of Sciences, Engineering, and Medicine established an international committee to discuss important issues about human genome editing and noted that somatic cell genome editing was expected to have the most immediate clinical application, but should only be used to treat or prevent disease and disability in accordance with current ethical standards (Human Genome Editing, 2017). In 2020, a consensus from International Commission on the Clinical Use of Human Germline Genome Editing defined translational pathway of heritable human genome editing to clinical use, and classified its potential application categories. According to the report, RH would fit in category B and suitable for using heritable human genome editing under stringent governance because of disease-causing FH allele both carried by prospective parents (Heritable Human Genome Editing, 2020). Besides, with current guidelines recommending that lower LDL-C is better (Grundy et al., 2019; Mach et al., 2020), gene editing is expected to further reduce LDL-C levels and improve the prognosis of RH patients, especially for those with genetic defects, or those who have had poor results or poor adherence with other treatment.

Another consideration is the cost gap between current therapies and gene therapy. Most studies have reported that gene therapy products were more costly than their comparators (Lin et al., 2018; Cher et al., 2020; Furzer et al., 2020). For example, Strimvelis^®^, a gene therapy treatment for severe combined immunodeficiency, will cause the incremental cost-effectiveness ratios when compared with hematopoietic stem cell transplants (hematopoetic stem cell transplant-haploidentical donor or hematopoietic stem cell transplant-matched unrelated donor) about £36,360 and £14,645 per quality-adjusted life-year, respectively (South et al., 2019). Although with the higher cost, potentially curative gene therapy offers the possibility of lifelong benefits from a single course of treatment, which could lead to these therapies being more cost-effective than current treatments in the long term (Ho et al., 2021). Therefore, the cost-effectiveness of gene therapy should be carefully considered from clinical trials to clinical practice (Jayasundera et al., 2022).

## Other New Targets for Cholesterol Reduction

Genome wide association study (GWAS) have identified nearly 80 genes with pathogenic variants to be associated with hypercholesterolemia, such as genes encoding LDLR, APOB, PCSK9, APOE, LDLRAP1, and the signal-transducing adaptor family member 1 (STAP1), among which mutations in LDLR account for most cases with FH (∼85–90%) (Paththinige et al., 2017; Di Taranto et al., 2020; Jackson et al., 2021). Several targets involved in lipid metabolism are already popular for lipid-regulating therapies, such as HMGCR (statins), NPC1L1 (ezetimibe), PCSK9 (alirocumab, evolocumab, inclisiran), and ANGPTL3 (evinacumab) (Raal et al., 2020b). Recently, novel targets have been recognized to act important function on cholesterol metabolism.

### REV-ERB

REV-ERB α, a member of the orphan nuclear hormone receptor superfamily, is one of the ligands activated transcription factors, and its isoform is REV-ERB β(Nr1D2), both of which are rhythmically expressed in supraoptic nucleus, liver and heart tissue. The heme-regulated nuclear receptor can regulate metabolic pathways. Previous studies have shown that treatment with synthetic REV-ERB agonists can inhibit plasma cholesterol levels and liver cholesterol biosynthesis rate-limiting enzyme (HMG CoA reductase) levels in mice. Animal studies have shown that the REV-ERB agonist SR9009 reduced plasma cholesterol levels in wild-type C57Bl/6 and LDLR deletion mice and reduced the expression of a series of genes in the cholesterol biosynthesis pathway. Consistent with these data, increased expression of these genes has been observed in mice with under-expression of REV-ERB α. Analysis showed that REV-ERB binds directly to most of the genes involved in cholesterol biosynthesis and directly inhibits their expression. This study reveals the complex mechanism by which REV-ERB regulates cholesterol biosynthesis directly or indirectly (by inhibiting Srebf2 expression) and provides information on how cholesterol levels are regulated in a circadian manner. This study suggests that targeting REV-ERB may be an effective method to reduce LDL-C levels clinically (Sitaula et al., 2017).

### G Protein-Coupled Receptor

Through bioinformatics analysis and functional verification, Han et al. recently found that SNPs rs1997243 was the only pathogenic mutation in the 7p22 region of the genome, which located in the non-coding region of the genome and specifically increased the expression of GPR146. Further study revealed that GPR146 encodes a G-protein-coupled receptor (GPCR), which is located in the plasma membrane of liver cells and activates the camp-PKA-CREb signaling pathway in response to serum stimulation, thereby regulating the balance of liver lipid metabolism and blood cholesterol level. This is the first GPCR that directly regulates blood cholesterol level. GPCR has good drug-forming properties, and more than 1/3 of all the drugs on the market are targeted by GPCR. This study provides a new important target for the development of cholesterol-lowering drugs (Han et al., 2020).

### Ubiquitin Specific Peptidase 20

Lu et al. first found that the protein content of the rate-limiting enzyme HMGCR in the cholesterol synthesis pathway significantly increased after eating. Using a cleverly designed *in vitro* biochemical reaction, they cloned and expressed over 70 deubiquitination enzymes one by one, and screened USP20 as hMGCr-specific deubiquitination enzyme. In the feeding state, the deubiquitination enzyme ubiquitin specific peptidase 20 (USP20) stabilized HMG-CoA reductase (HMGCR), a rate-limiting enzyme in the cholesterol biosynthesis pathway. Postprandial increases in insulin and glucose concentrations synergistically activated mTORC1 and phosphorylate USP20 at S132 and S134. USP20 was recruited to the HMGCR complex and antagonized its degradation, up-regulating cholesterol synthesis and converting absorbed glucose and other nutrients into cholesterol. Subsequently, it was also found that long-term high-sugar and high-fat diet induced increased phosphorylation of USP20, stabilized HMGCR protein and increased cholesterol, which caused metabolic diseases. In order to explore whether USP20 can be used as a therapeutic target for metabolic diseases such as obesity, investigators gave USP20 inhibitor to obese mice. They found that USP20 inhibitor can significantly reduce body weight, reduce blood cholesterol and triglyceride levels, and improve insulin sensitivity. Inhibition of USP20 can promote HMGCR degradation and decrease lipid synthesis. It also caused succinic acid to increase and increased heat production. The improvement of these metabolic indicators is conducive to the treatment of metabolic diseases such as hypercholesterolemia, obesity and diabetes (Lu et al., 2020).

## Conclusion

In summary, research on therapeutic targets for refractory hypercholesterolemia is being carried out in depth. At the same time, for the treatment of refractory hypercholesterolemia, gene therapy technology is still developing, and is expected to achieve new breakthroughs. In view of these innovative techniques/approaches, more research efforts are currently focused on developing precise, effective and safe gene delivery and editing strategies for genetic therapy of RH. However, potential risks of novel drugs should always be carefully considered, especially at the aspect of human genome editing. Therefore, further studies are needed to induce effective RH-related gene transgenic expression safely, balance the risks and benefits of clinical use, and ultimately achieve sustained reduction and regression of atherosclerosis in humans. Another consideration is the higher cost potential lifelong benefits caused by gene therapy. Therefore, the cost-effectiveness of gene therapy need to be carefully considered. Gene therapy has always been a research hotspot in the field of RH, and it is committed to developing effective and safe treatment methods that can be successfully transformed into clinical application. In addition, new targets such as REV-ERB, GPCR and USP20 are expected to be used in the clinic in the near future. Although the initial results are promising, more, larger and longer clinical trials are needed to determine the exact role of these approaches in the treatment of refractory hypercholesterolemia.

## Abbreviations

RH, refractory hypercholesterolemia; HoFH, homozygous familial hypercholesterolemia; HeFH, heterozygous familial hypercholesterolemia; LDL-C, low-density lipoprotein cholesterol; ASCVD, atherosclerotic cardiovascular disease; PCSK9, proprotein convertase subtilisin-kexin type 9; ANGPTL3, angiopoietin-like protein 3; mAbs, monoclonal antibodies; Apo, apolipoprotein; siRNA, small interfering RNA; ASO, antisense oligonucleotide; VLDL, very-low-density lipoprotein; Lp(a), lipoprotein (a); Ad, adenovirus; AAV, adeno-associated virus; IDOL, inducible degrader of LDLR; hiPSC, human induced pluripotent stem cell; HMG-COA, 3-hydroxy3-methylglutarate reductase; LNP, Lipid nanoparticle; sgRNA, single guide RNA; CRISPR, Clustering regularly spaced short palindromic repeats; Cas, CRISPR-associated protein; SaCas9, *staphylococcus aureus* Cas9; NmeCas9, neisseria meningitidis Cas9; HLC, hepatocellular like cell; indel, insertion and deletion; CBE, cytosine base-editor; ABE, adenine base-editor; BE3, the third-generation base editor; GPCR, G protein-coupled receptor; USP20, ubiquitin specific peptidase 20.

## References

[B1] Aguilar-Salinas C. A. Gómez-Díaz R. A. Corral P. (2021). New Therapies for Primary Hyperlipidemia. J. Clin. Endocrinol. Metab. 107, 1216–1224. 10.1210/clinem/dgab876 34888679

[B2] Akoumianakis I. Zvintzou E. Kypreos K. Filippatos T. D. (2021). Angptl3 and Apolipoprotein C-Iii as Novel Lipid-Lowering Targets. Curr. Atheroscler. Rep. 23 (5), 20. 10.1007/s11883-021-00914-7 33694000

[B3] Andreoletti M. Loux N. Vons C. Nguyen T. H. Lorand I. Mahieu D. (2001). Engraftment of Autologous Retrovirally Transduced Hepatocytes after Intraportal Transplantation into Nonhuman Primates: Implication Forex VivoGene Therapy. Hum. Gene Ther. 12 (2), 169–179. 10.1089/104303401750061230 11177554

[B4] Anzalone A. V. Koblan L. W. Liu D. R. (2020). Genome Editing with Crispr-Cas Nucleases, Base Editors, Transposases and Prime Editors. Nat. Biotechnol. 38 (7), 824–844. 10.1038/s41587-020-0561-9 32572269

[B5] Arrowhead Reports Interim Clinical Data on Cardiometabolic Candidates Aro-Apoc3 and Aro-Ang3 Arrowhead Reports Interim Clinical Data on Cardiometabolic Candidates Aro-Apoc3 and Aro-Ang3. (2020):2.

[B6] Austin M. A. Hutter C. M. Zimmern R. L. Humphries S. E. (2004). Genetic Causes of Monogenic Heterozygous Familial Hypercholesterolemia: A Huge Prevalence Review. Am. J. Epidemiol. 160 (5), 407–420. 10.1093/aje/kwh236 15321837

[B7] Baek G. Choi H. Kim Y. Lee H.-C. Choi C. (2019). Mesenchymal Stem Cell-Derived Extracellular Vesicles as Therapeutics and as a Drug Delivery Platform. Stem Cells Transl. Med. 8 (9), 880–886. 10.1002/sctm.18-0226 31045328 PMC6708072

[B8] Beheshti S. O. Madsen C. M. Varbo A. Nordestgaard B. G. (2020). Worldwide Prevalence of Familial Hypercholesterolemia. J. Am. Coll. Cardiol. 75 (20), 2553–2566. 10.1016/j.jacc.2020.03.057 32439005

[B9] Bell D. A. Watts G. F. (2016). Progress in the Care of Familial Hypercholesterolaemia: 2016. Med. J. Aust. 205 (5), 232–236. 10.5694/mja16.00070 27581271

[B10] Bellosta S. Rossi C. Alieva A. S. Catapano A. L. Corsini A. Baragetti A. (2022). Cholesterol Lowering Biotechnological Strategies: From Monoclonal Antibodies to Antisense Therapies. A Pre-clinical Perspective Review. Cardiovasc Drugs Ther. 10.1007/s10557-021-07293-w 35022949

[B11] Blom D. J. Fayad Z. A. Kastelein J. J. P. Larrey D. Makris L. Schwamlein C. (2016). Lower, a Registry of Lomitapide-Treated Patients with Homozygous Familial Hypercholesterolemia: Rationale and Design. J. Clin. Lipidol. 10 (2), 273–282. 10.1016/j.jacl.2015.11.011 27055957

[B12] Bolukbasi M. F. Gupta A. Wolfe S. A. (2016). Creating and Evaluating Accurate Crispr-Cas9 Scalpels for Genomic Surgery. Nat. Methods 13 (1), 41–50. 10.1038/nmeth.3684 26716561

[B13] Brandts J. Ray K. K. (2020). Bempedoic Acid, an Inhibitor of Atp Citrate Lyase for the Treatment of Hypercholesterolemia: Early Indications and Potential. Expert Opin. Investigational Drugs 29 (8), 763–770. 10.1080/13543784.2020.1778668 32564642

[B14] Brandts J. Ray K. K. (2021). Familial Hypercholesterolemia. J. Am. Coll. Cardiol. 78 (18), 1831–1843. 10.1016/j.jacc.2021.09.004 34711342

[B15] Cao G. Xuan X. Zhang R. Hu J. Dong H. (2021). Gene Therapy for Cardiovascular Disease: Basic Research and Clinical Prospects. Front. Cardiovasc. Med. 8, 760140. 10.3389/fcvm.2021.760140 34805315 PMC8602679

[B16] Carreras A. Pane L. S. Nitsch R. Madeyski-Bengtson K. Porritt M. Akcakaya P. (2019). *In Vivo* genome and Base Editing of a Human PCSK9 Knock-In Hypercholesterolemic Mouse Model. BMC Biol. 17 (1), 4. 10.1186/s12915-018-0624-2 30646909 PMC6334452

[B17] Chadwick A. C. Evitt N. H. Lv W. Musunuru K. (2018). Reduced Blood Lipid Levels with *In Vivo* Crispr-Cas9 Base Editing of Angptl3. Circulation 137 (9), 975–977. 10.1161/CIRCULATIONAHA.117.031335 29483174 PMC5830171

[B18] Chadwick A. C. Wang X. Musunuru K. (2017). *In Vivo* Base Editing of PCSK9 (Proprotein Convertase Subtilisin/Kexin Type 9) as a Therapeutic Alternative to Genome Editing. Atvb 37 (9), 1741–1747. 10.1161/ATVBAHA.117.309881 PMC5570639 28751571

[B19] Chambergo-Michilot D. Alur A. Kulkarni S. Agarwala A. (2022). Mipomersen in Familial Hypercholesterolemia: An Update on Health-Related Quality of Life and Patient-Reported Outcomes. Vhrm Vol. 18, 73–80. 10.2147/VHRM.S191965 PMC8880726 35221690

[B20] Cher B. P. Gan K. Y. Aziz M. I. A. Lin L. Hwang W. Y. K. Poon L. M. (2020). Cost Utility Analysis of Tisagenlecleucel vs Salvage Chemotherapy in the Treatment of Relapsed/Refractory Diffuse Large B-Cell Lymphoma from Singapore's Healthcare System Perspective. J. Med. Econ. 23 (11), 1321–1329. 10.1080/13696998.2020.1808981 32780608

[B21] Chi X. Gatti P. Papoian T. (2017). Safety of Antisense Oligonucleotide and Sirna-Based Therapeutics. Drug Discov. Today 22 (5), 823–833. 10.1016/j.drudis.2017.01.013 28159625

[B22] Conway A. Mendel M. Kim K. McGovern K. Boyko A. Zhang L. (2019). Non-Viral Delivery of Zinc Finger Nuclease Mrna Enables Highly Efficient *In Vivo* Genome Editing of Multiple Therapeutic Gene Targets. Mol. Ther. 27 (4), 866–877. 10.1016/j.ymthe.2019.03.003 30902585 PMC6453547

[B23] Cuchel M. Bruckert E. Ginsberg H. N. Raal F. J. Santos R. D. Hegele R. A. (2014). Homozygous Familial Hypercholesterolaemia: New Insights and Guidance for Clinicians to Improve Detection and Clinical Management. A Position Paper from the Consensus Panel on Familial Hypercholesterolaemia of the European Atherosclerosis Society. Eur. Heart J. 35 (32), 2146–2157. 10.1093/eurheartj/ehu274 25053660 PMC4139706

[B24] D’Erasmo L. Cefalù A. B. Noto D. Giammanco A. Averna M. Pintus P. (2017). Efficacy of Lomitapide in the Treatment of Familial Homozygous Hypercholesterolemia: Results of a Real-World Clinical Experience in Italy. Adv. Ther. 34 (5), 1200–1210. 10.1007/s12325-017-0531-x 28432645

[B25] Di Taranto M. D. Giacobbe C. Fortunato G. (2020). Familial Hypercholesterolemia: A Complex Genetic Disease with Variable Phenotypes. Eur. J. Med. Genet. 63 (4), 103831. 10.1016/j.ejmg.2019.103831 31883481

[B26] Ding Q. Strong A. Patel K. M. Ng S.-L. Gosis B. S. Regan S. N. (2014). Permanent Alteration of Pcsk9 with *In Vivo* Crispr-Cas9 Genome Editing. Circ. Res. 115 (5), 488–492. 10.1161/CIRCRESAHA.115.304351 24916110 PMC4134749

[B27] Doudna J. A. (2020). The Promise and Challenge of Therapeutic Genome Editing. Nature 578 (7794), 229–236. 10.1038/s41586-020-1978-5 32051598 PMC8992613

[B29] Esvelt K. M. Mali P. Braff J. L. Moosburner M. Yaung S. J. Church G. M. (2013). Orthogonal Cas9 Proteins for Rna-Guided Gene Regulation and Editing. Nat. Methods 10 (11), 1116–1121. 10.1038/nmeth.2681 24076762 PMC3844869

[B30] Fu Y. Foden J. A. Khayter C. Maeder M. L. Reyon D. Joung J. K. (2013). High-Frequency Off-Target Mutagenesis Induced by Crispr-Cas Nucleases in Human Cells. Nat. Biotechnol. 31 (9), 822–826. 10.1038/nbt.2623 23792628 PMC3773023

[B31] Furzer J. Gupta S. Nathan P. C. Schechter T. Pole J. D. Krueger J. (2020). Cost-Effectiveness of Tisagenlecleucel vs Standard Care in High-Risk Relapsed Pediatric Acute Lymphoblastic Leukemia in Canada. JAMA Oncol. 6 (3), 393–401. 10.1001/jamaoncol.2019.5909 31971547 PMC6990832

[B32] Graham M. J. Lee R. G. Brandt T. A. Tai L.-J. Fu W. Peralta R. (2017). Cardiovascular and Metabolic Effects of Angptl3 Antisense Oligonucleotides. N. Engl. J. Med. 377 (3), 222–232. 10.1056/NEJMoa1701329 28538111

[B33] Greig J. A. Limberis M. P. Bell P. Chen S.-J. Calcedo R. Rader D. J. (2017). Non-Clinical Study Examining AAV8.TBG.hLDLR Vector-Associated Toxicity in Chow-Fed Wild-type and LDLR+/− Rhesus Macaques. Hum. Gene Ther. Clin. Dev. 28 (1), 39–50. 10.1089/humc.2017.014 28319449 PMC5369385

[B34] Greig J. A. Limberis M. P. Bell P. Chen S.-J. Calcedo R. Rader D. J. (2017). Nonclinical Pharmacology/Toxicology Study of Aav8.Tbg.Mldlr and Aav8.Tbg.Hldlr in a Mouse Model of Homozygous Familial Hypercholesterolemia. Hum. Gene Ther. Clin. Dev. 28 (1), 28–38. 10.1089/humc.2017.007 28319445 PMC5369398

[B35] Grossman M. Rader D. J. Muller D. W. M. Kolansky D. M. Kozarsky K. Clark B. J. 3rd (1995). A Pilot Study of *Ex Vivo* Gene Therapy for Homozygous Familial Hypercholesterolaemia. Nat. Med. 1 (11), 1148–1154. 10.1038/nm1195-1148 7584986

[B36] Grundy S. M. Stone N. J. Bailey A. L. Beam C. Birtcher K. K. Blumenthal R. S. (2019). 2018 Aha/Acc/Aacvpr/Aapa/Abc/Acpm/Ada/Ags/Apha/Aspc/Nla/Pcna Guideline on the Management of Blood Cholesterol: A Report of the American College of Cardiology/American Heart Association Task Force on Clinical Practice Guidelines. Circulation 139 (25), e1082–e143. 10.1161/CIR.0000000000000625 30586774 PMC7403606

[B37] Gupta A. Andresen J. L. Manan R. S. Langer R. (2021). Nucleic Acid Delivery for Therapeutic Applications. Adv. Drug Deliv. Rev. 178, 113834. 10.1016/j.addr.2021.113834 34492233

[B38] Han F. Liu X. Chen C. Liu Y. Du M. Zhou Y. (2020). Hypercholesterolemia Risk-Associated Gpr146 Is an Orphan G-Protein Coupled Receptor that Regulates Blood Cholesterol Levels in Humans and Mice. Cell. Res. 30 (4), 363–365. 10.1038/s41422-020-0303-z 32203133 PMC7118070

[B39] Hartgers M. L. Defesche J. C. Langslet G. Hopkins P. N. Kastelein J. J. P. Baccara-Dinet M. T. (2018). Alirocumab Efficacy in Patients with Double Heterozygous, Compound Heterozygous, or Homozygous Familial Hypercholesterolemia. J. Clin. Lipidol. 12 (2), 390–396. 10.1016/j.jacl.2017.12.008 29396260

[B40] Hartgers M. L. Ray K. K. Hovingh G. K. (2015). New Approaches in Detection and Treatment of Familial Hypercholesterolemia. Curr. Cardiol. Rep. 17 (12), 109. 10.1007/s11886-015-0665-x 26482752 PMC4611021

[B41] Henry S. P. Narayanan P. Shen L. Bhanot S. Younis H. S. Burel S. A. (2017). Assessment of the Effects of 2′-Methoxyethyl Antisense Oligonucleotides on Platelet Count in Cynomolgus Nonhuman Primates. Nucleic Acid. Ther. 27 (4), 197–208. 10.1089/nat.2017.0666 28541820

[B42] Heritable Human Genome Editing. The National Academies Collection: Reports Funded by National Institutes of Health. Washington (DC)(2020).

[B43] Ho J. K. Borle K. Dragojlovic N. Dhillon M. Kitchin V. Kopac N. (2021). Economic Evidence on Potentially Curative Gene Therapy Products: A Systematic Literature Review. Pharmacoeconomics 39 (9), 995–1019. 10.1007/s40273-021-01051-4 34156648

[B44] Hou X. Jiao R. Guo X. Wang T. Chen P. Wang D. (2016). Construction of Minicircle DNA Vectors Capable of Correcting Familial Hypercholesterolemia Phenotype in a Ldlr-Deficient Mouse Model. Gene Ther. 23 (8-9), 657–663. 10.1038/gt.2016.37 27092942

[B45] Human Genome Editing: Science, Ethics, and Governance. Washington (DC)(2017). 28796468

[B46] Hytönen E. Laurema A. Kankkonen H. Miyanohara A. Kärjä V. Hujo M. (2019). Bile-Duct Proliferation as an Unexpected Side-Effect after Aav2-Ldlr Gene Transfer to Rabbit Liver. Sci. Rep. 9 (1), 6934. 10.1038/s41598-019-43459-1 31061510 PMC6502883

[B47] Ibraheim R. Song C.-Q. Mir A. Amrani N. Xue W. Sontheimer E. J. (2018). All-in-One Adeno-Associated Virus Delivery and Genome Editing by Neisseria Meningitidis Cas9 *In Vivo* . Genome Biol. 19 (1), 137. 10.1186/s13059-018-1515-0 30231914 PMC6146650

[B48] Ibrahim S. Somanathan S. Billheimer J. Wilson J. M. Rader D. J. (2016). Stable Liver-specific Expression of Human Idol in Humanized Mice Raises Plasma Cholesterol. Cardiovasc Res. 110 (1), 23–29. 10.1093/cvr/cvw010 26786161 PMC4798044

[B49] Jackson C. L. Zordok M. Kullo I. J. (2021). Familial Hypercholesterolemia in Southeast and East Asia. Am. J. Prev. Cardiol. 6, 100157. 10.1016/j.ajpc.2021.100157 34327494 PMC8315601

[B50] Jayasundera K. T. Abuzaitoun R. O. Lacy G. D. Abalem M. F. Saltzman G. M. Ciulla T. A. (2022). Challenges of Cost-Effectiveness Analyses of Novel Therapeutics for Inherited Retinal Diseases. Am. J. Ophthalmol. 235, 90–97. 10.1016/j.ajo.2021.08.009 34433085 PMC8861129

[B51] Jiang C. Mei M. Li B. Zhu X. Zu W. Tian Y. (2017). A Non-viral Crispr/Cas9 Delivery System for Therapeutically Targeting Hbv DNA and Pcsk9 *In Vivo* . Cell. Res. 27 (3), 440–443. 10.1038/cr.2017.16 28117345 PMC5339835

[B52] Kantor A. McClements M. MacLaren R. (2020). Crispr-Cas9 DNA Base-Editing and Prime-Editing. Ijms 21 (17), 6240. 10.3390/ijms21176240 PMC7503568 32872311

[B53] Kerr A. G. Tam L. C. Hale A. B. Cioroch M. Douglas G. Channon K. M. (2016). Episomal Nonviral Gene Therapy Vectors Slow Progression of Atherosclerosis in a Model of Familial Hypercholesterolemia. Mol. Ther. - Nucleic Acids 5 (11), e383. 10.1038/mtna.2016.86 27824334 PMC5155321

[B54] Khoo B. (2015). Genetic Therapies to Lower Cholesterol. Vasc. Pharmacol. 64, 11–15. 10.1016/j.vph.2014.12.002 25542072

[B55] Kim D. Lim K. Kim S.-T. Yoon S.-h. Kim K. Ryu S.-M. (2017). Genome-Wide Target Specificities of Crispr Rna-Guided Programmable Deaminases. Nat. Biotechnol. 35 (5), 475–480. 10.1038/nbt.3852 28398345

[B56] Kim E. Koo T. Park S. W. Kim D. Kim K. Cho H.-Y. (2017). *In Vivo* genome Editing with a Small Cas9 Orthologue Derived from Campylobacter Jejuni. Nat. Commun. 8, 14500. 10.1038/ncomms14500 28220790 PMC5473640

[B57] Komor A. C. Badran A. H. Liu D. R. (2018). Editing the Genome without Double-Stranded DNA Breaks. ACS Chem. Biol. 13 (2), 383–388. 10.1021/acschembio.7b00710 28957631 PMC5891729

[B58] Kuijper E. C. Bergsma A. J. Pijnappel W. W. M. P. Aartsma‐Rus A. (2021). Opportunities and Challenges for Antisense Oligonucleotide Therapies. Jrnl Inher Metab Disea 44 (1), 72–87. 10.1002/jimd.12251 PMC7891411 32391605

[B59] Lau C. H. Suh Y. (2017). *In Vivo* genome Editing in Animals Using AAV-CRISPR System: Applications to Translational Research of Human Disease. F1000Res 6, F1000Res2153. 10.12688/f1000research.11243.1 PMC5749125 29333255

[B60] Lebherz C. Gao G. Louboutin J.-P. Millar J. Rader D. Wilson J. M. (2004). Gene Therapy with Novel Adeno-Associated Virus Vectors Substantially Diminishes Atherosclerosis in a Murine Model of Familial Hypercholesterolemia. J. Gene Med. 6 (6), 663–672. 10.1002/jgm.554 15170737

[B61] Leggiero E. Astone D. Cerullo V. Lombardo B. Mazzaccara C. Labruna G. (2013). Pegylated Helper-dependent Adenoviral Vector Expressing Human Apo A-I for Gene Therapy in Ldlr-Deficient Mice. Gene Ther. 20 (12), 1124–1130. 10.1038/gt.2013.38 23883962

[B62] Leggiero E. Labruna G. Iaffaldano L. Lombardo B. Greco A. Fiorenza D. (2019). Helper-Dependent Adenovirus-Mediated Gene Transfer of a Secreted Ldl Receptor/Transferrin Chimeric Protein Reduces Aortic Atherosclerosis in Ldl Receptor-Deficient Mice. Gene Ther. 26 (3-4), 121–130. 10.1038/s41434-019-0061-z 30700805

[B63] Li J.-J. Li S. Zhu C.-G. Wu N.-Q. Zhang Y. Guo Y.-L. (2017). Familial Hypercholesterolemia Phenotype in Chinese Patients Undergoing Coronary Angiography. Atvb 37 (3), 570–579. 10.1161/ATVBAHA.116.308456 27932355

[B64] Li N. Li Q. Tian X.-Q. Qian H.-Y. Yang Y.-J. (2014). Mipomersen Is a Promising Therapy in the Management of Hypercholesterolemia: A Meta-Analysis of Randomized Controlled Trials. Am. J. Cardiovasc Drugs 14 (5), 367–376. 10.1007/s40256-014-0077-0 25027352

[B65] Li S. Zhang H.-W. Guo Y.-L. Wu N.-Q. Zhu C.-G. Zhao X. (2018). Familial Hypercholesterolemia in Very Young Myocardial Infarction. Sci. Rep. 8 (1), 8861. 10.1038/s41598-018-27248-w 29892007 PMC5995844

[B66] Li S. Zhang Y. Zhu C.-G. Guo Y.-L. Wu N.-Q. Gao Y. (2016). Identification of Familial Hypercholesterolemia in Patients with Myocardial Infarction: A Chinese Cohort Study. J. Clin. Lipidol. 10 (6), 1344–1352. 10.1016/j.jacl.2016.08.013 27919351

[B67] Li Z. Zhao P. Zhang Y. Wang J. Wang C. Liu Y. (2021). Exosome-Based Ldlr Gene Therapy for Familial Hypercholesterolemia in a Mouse Model. Theranostics 11 (6), 2953–2965. 10.7150/thno.49874 33456582 PMC7806494

[B68] Liang P. Xie X. Zhi S. Sun H. Zhang X. Chen Y. (2019). Genome-Wide Profiling of Adenine Base Editor Specificity by Endov-Seq. Nat. Commun. 10 (1), 67. 10.1038/s41467-018-07988-z 30622278 PMC6325126

[B69] Lin J. K. Lerman B. J. Barnes J. I. Boursiquot B. C. Tan Y. J. Robinson A. Q. L. (2018). Cost Effectiveness of Chimeric Antigen Receptor T-Cell Therapy in Relapsed or Refractory Pediatric B-Cell Acute Lymphoblastic Leukemia. Jco 36 (32), 3192–3202. 10.1200/jco.2018.79.0642 30212291

[B70] Liu G. Lin Q. Jin S. Gao C. (2022). The Crispr-Cas Toolbox and Gene Editing Technologies. Mol. Cell. 82 (2), 333–347. 10.1016/j.molcel.2021.12.002 34968414

[B71] Lu X.-Y. Shi X.-J. Hu A. Wang J.-Q. Ding Y. Jiang W. (2020). Feeding Induces Cholesterol Biosynthesis via the Mtorc1-Usp20-Hmgcr Axis. Nature 588 (7838), 479–484. 10.1038/s41586-020-2928-y 33177714

[B72] Mach F. Baigent C. Catapano A. L. Koskinas K. C. Casula M. Badimon L. (2020). 2019 ESC/EAS Guidelines for the Management of Dyslipidaemias: Lipid Modification to Reduce Cardiovascular Risk. Eur. Heart J. 41 (1), 111–188. 10.1093/eurheartj/ehz455 31504418

[B73] Maczuga P. Verheij J. van der Loos C. van Logtenstein R. Hooijer G. Martier R. (2014). Therapeutic Expression of Hairpins Targeting Apolipoprotein B100 Induces Phenotypic and Transcriptome Changes in Murine Liver. Gene Ther. 21 (1), 60–70. 10.1038/gt.2013.58 24152580 PMC3881031

[B74] Maestro S. Weber N. D. Zabaleta N. Aldabe R. Gonzalez-Aseguinolaza G. (2021). Novel Vectors and Approaches for Gene Therapy in Liver Diseases. JHEP Rep. 3 (4), 100300. 10.1016/j.jhepr.2021.100300 34159305 PMC8203845

[B75] Mendell J. R. Al-Zaidy S. Shell R. Arnold W. D. Rodino-Klapac L. R. Prior T. W. (2017). Single-Dose Gene-Replacement Therapy for Spinal Muscular Atrophy. N. Engl. J. Med. 377 (18), 1713–1722. 10.1056/NEJMoa1706198 29091557

[B76] Mohamed F. Botha T. C. Raal F. J. (2021). Inhibition of Angiopoietin-like 3 for the Management of Severe Hypercholesterolemia. Curr. Opin. Lipidol. Publish Ahead of Print (4), 213–218. 10.1097/MOL.0000000000000755 33883446

[B77] Musunuru K. Chadwick A. C. Mizoguchi T. Garcia S. P. DeNizio J. E. Reiss C. W. (2021). *In Vivo* CRISPR Base Editing of PCSK9 Durably Lowers Cholesterol in Primates. Nature 593 (7859), 429–434. 10.1038/s41586-021-03534-y 34012082

[B78] Musunuru K. Pirruccello J. P. Do R. Peloso G. M. Guiducci C. Sougnez C. (2010). Exome Sequencing,ANGPTL3Mutations, and Familial Combined Hypolipidemia. N. Engl. J. Med. 363 (23), 2220–2227. 10.1056/NEJMoa1002926 20942659 PMC3008575

[B79] Nomura S. Merched A. Nour E. Dieker C. Oka K. Chan L. (2004). Low-Density Lipoprotein Receptor Gene Therapy Using Helper-dependent Adenovirus Produces Long-Term Protection against Atherosclerosis in a Mouse Model of Familial Hypercholesterolemia. Gene Ther. 11 (20), 1540–1548. 10.1038/sj.gt.3302310 15269711

[B80] Oertel M. Rosencrantz R. Chen Y. Q. Thota P. N. Sandhu J. S. Dabeva M. D. (2003). Repopulation of Rat Liver by Fetal Hepatoblasts and Adult Hepatocytes Transduced *Ex Vivo* with Lentiviral Vectors. Hepatology 37 (5), 994–1005. 10.1053/jhep.2003.50183 12717380

[B81] Okada H. Nakanishi C. Yoshida S. Shimojima M. Yokawa J. Mori M. (2019). Function and Immunogenicity of Gene-Corrected Ipsc-Derived Hepatocyte-like Cells in Restoring Low Density Lipoprotein Uptake in Homozygous Familial Hypercholesterolemia. Sci. Rep. 9 (1), 4695. 10.1038/s41598-019-41056-w 30886174 PMC6423040

[B82] Ou H. Zhang Q. Zeng J. (2016). Improving Lipoprotein Profiles by Liver-Directed Gene Transfer of Low Density Lipoprotein Receptor Gene in Hypercholesterolaemia Mice. J. Genet. 95 (2), 311–316. 10.1007/s12041-016-0636-z 27350674

[B83] Paththinige C. Sirisena N. Dissanayake V. (2017). Genetic Determinants of Inherited Susceptibility to Hypercholesterolemia - a Comprehensive Literature Review. Lipids Health Dis. 16 (1), 103. 10.1186/s12944-017-0488-4 28577571 PMC5457620

[B84] Pennesi M. E. Weleber R. G. Yang P. Whitebirch C. Thean B. Flotte T. R. (2018). Results at 5 Years after Gene Therapy for Rpe65-Deficient Retinal Dystrophy. Hum. Gene Ther. 29 (12), 1428–1437. 10.1089/hum.2018.014 29869534 PMC12199623

[B85] Porteus M. H. (2019). A New Class of Medicines through DNA Editing. N. Engl. J. Med. 380 (10), 947–959. 10.1056/NEJMra1800729 30855744

[B86] Raal F. J. Kallend D. Ray K. K. Turner T. Koenig W. Wright R. S. (2020). Inclisiran for the Treatment of Heterozygous Familial Hypercholesterolemia. N. Engl. J. Med. 382 (16), 1520–1530. 10.1056/NEJMoa1913805 32197277

[B87] Raal F. J. Rosenson R. S. Reeskamp L. F. Hovingh G. K. Kastelein J. J. P. Rubba P. (2020). Evinacumab for Homozygous Familial Hypercholesterolemia. N. Engl. J. Med. 383 (8), 711–720. 10.1056/NEJMoa2004215 32813947

[B88] Raal F. J. Santos R. D. Blom D. J. Marais A. D. Charng M.-J. Cromwell W. C. (2010). Mipomersen, an Apolipoprotein B Synthesis Inhibitor, for Lowering of Ldl Cholesterol Concentrations in Patients with Homozygous Familial Hypercholesterolaemia: A Randomised, Double-Blind, Placebo-Controlled Trial. Lancet 375 (9719), 998–1006. 10.1016/S0140-6736(10)60284-X 20227758

[B89] Rached F. Santos R. D. (2021). Beyond Statins and Pcsk9 Inhibitors: Updates in Management of Familial and Refractory Hypercholesterolemias. Curr. Cardiol. Rep. 23 (7), 83. 10.1007/s11886-021-01514-2 34081216

[B90] Ran F. A. Cong L. Yan W. X. Scott D. A. Gootenberg J. S. Kriz A. J. (2015). *In Vivo* genome Editing Using *Staphylococcus aureus* Cas9. Nature 520 (7546), 186–191. 10.1038/nature14299 25830891 PMC4393360

[B91] Ray K. K. Wright R. S. Kallend D. Koenig W. Leiter L. A. Raal F. J. (2020). Two Phase 3 Trials of Inclisiran in Patients with Elevated Ldl Cholesterol. N. Engl. J. Med. 382 (16), 1507–1519. 10.1056/NEJMoa1912387 32187462

[B92] Rees H. A. Liu D. R. (2018). Base Editing: Precision Chemistry on the Genome and Transcriptome of Living Cells. Nat. Rev. Genet. 19 (12), 770–788. 10.1038/s41576-018-0059-1 30323312 PMC6535181

[B93] Rosenson R. S. Burgess L. J. Ebenbichler C. F. Baum S. J. Stroes E. S. G. Ali S. (2020). Evinacumab in Patients with Refractory Hypercholesterolemia. N. Engl. J. Med. 383 (24), 2307–2319. 10.1056/NEJMoa2031049 33196153

[B94] Rothgangl T. Dennis M. K. Lin P. J. C. Oka R. Witzigmann D. Villiger L. (2021). *In Vivo* adenine Base Editing of PCSK9 in Macaques Reduces LDL Cholesterol Levels. Nat. Biotechnol. 39 (8), 949–957. 10.1038/s41587-021-00933-4 34012094 PMC8352781

[B95] Ruscica M. Zimetti F. Adorni M. P. Sirtori C. R. Lupo M. G. Ferri N. (2020). Pharmacological Aspects of Angptl3 and Angptl4 Inhibitors: New Therapeutic Approaches for the Treatment of Atherogenic Dyslipidemia. Pharmacol. Res. 153, 104653. 10.1016/j.phrs.2020.104653 31931117

[B96] Russell S. Bennett J. Wellman J. A. Chung D. C. Yu Z.-F. Tillman A. (2017). Efficacy and Safety of Voretigene Neparvovec (AAV2-hRPE65v2) in Patients with RPE65 -mediated Inherited Retinal Dystrophy: a Randomised, Controlled, Open-Label, Phase 3 Trial. Lancet 390 (10097), 849–860. 10.1016/S0140-6736(17)31868-8 28712537 PMC5726391

[B28] Sabatine M. S. (2019). PCSK9 Inhibitors: Clinical Evidence and Implementation. Nat. Rev. Cardiol. 16 (3), 155–165. 10.1038/s41569-018-0107-8 30420622

[B97] Sharma G. Sharma A. R. Bhattacharya M. Lee S.-S. Chakraborty C. (2021). Crispr-Cas9: A Preclinical and Clinical Perspective for the Treatment of Human Diseases. Mol. Ther. 29 (2), 571–586. 10.1016/j.ymthe.2020.09.028 33238136 PMC7854284

[B98] Shi H.-W. Yang J.-G. Wang Y. Li W. Guo Y.-L. Gao Y. (2020). The Prevalence of Familial Hypercholesterolemia (Fh) in Chinese Patients with Acute Myocardial Infarction (Ami): Data from Chinese Acute Myocardial Infarction (Cami) Registry. Front. Cardiovasc. Med. 7, 113. 10.3389/fcvm.2020.00113 32766283 PMC7378384

[B99] Singh A. K. Chaube B. Zhang X. Sun J. Citrin K. M. Canfrán-Duque A. (2021). Hepatocyte-Specific Suppression of Angptl4 Improves Obesity-Associated Diabetes and Mitigates Atherosclerosis in Mice. J. Clin. Invest. 131. 10.1172/JCI140989 PMC8409581 34255741

[B100] Sitaula S. Zhang J. Ruiz F. Burris T. P. (2017). Rev-Erb Regulation of Cholesterologenesis. Biochem. Pharmacol. 131, 68–77. 10.1016/j.bcp.2017.02.006 28213272 PMC9446480

[B101] Sjouke B. Kusters D. M. Kindt I. Besseling J. Defesche J. C. Sijbrands E. J. G. (2015). Homozygous Autosomal Dominant Hypercholesterolaemia in the Netherlands: Prevalence, Genotype-Phenotype Relationship, and Clinical Outcome. Eur. Heart J. 36 (9), 560–565. 10.1093/eurheartj/ehu058 24585268

[B102] Sjouke B. Langslet G. Ceska R. Nicholls S. J. Nissen S. E. Öhlander M. (2014). Eprotirome in Patients with Familial Hypercholesterolaemia (The Akka Trial): A Randomised, Double-Blind, Placebo-Controlled Phase 3 Study. Lancet Diabetes & Endocrinol. 2 (6), 455–463. 10.1016/S2213-8587(14)70006-3 24731671

[B103] South E. Cox E. Meader N. Woolacott N. Griffin S. (2019). Strimvelis for Treating Severe Combined Immunodeficiency Caused by Adenosine Deaminase Deficiency: An Evidence Review Group Perspective of a NICE Highly Specialised Technology Evaluation. PharmacoEconomics Open 3 (2), 151–161. 10.1007/s41669-018-0102-3 30334168 PMC6533345

[B104] Tardif J.-C. Karwatowska-Prokopczuk E. Amour E. S. Ballantyne C. M. Shapiro M. D. Moriarty P. M. (2022). Apolipoprotein C-Iii Reduction in Subjects with Moderate Hypertriglyceridaemia and at High Cardiovascular Risk. Eur. Heart J. 43 (14), 1401–1412. 10.1093/eurheartj/ehab820 35025993 PMC8986458

[B105] Wang L. Breton C. Warzecha C. C. Bell P. Yan H. He Z. (2021). Long-Term Stable Reduction of Low-Density Lipoprotein in Nonhuman Primates Following *In Vivo* Genome Editing of Pcsk9. Mol. Ther. 29 (6), 2019–2029. 10.1016/j.ymthe.2021.02.020 33609733 PMC8178442

[B106] Wang L. Smith J. Breton C. Clark P. Zhang J. Ying L. (2018). Meganuclease Targeting of Pcsk9 in Macaque Liver Leads to Stable Reduction in Serum Cholesterol. Nat. Biotechnol. 36 (8), 717–725. 10.1038/nbt.4182 29985478

[B107] Wang X. Raghavan A. Chen T. Qiao L. Zhang Y. Ding Q. (2016). Crispr-Cas9 Targeting of Pcsk9 in Human Hepatocytes In Vivo-Brief Report. Atvb 36 (5), 783–786. 10.1161/ATVBAHA.116.307227 PMC4850082 26941020

[B108] Watts G. F. Schwabe C. Scott R. Gladding P. Sullivan D. Baker J. (2020). Pharmacodynamic Effect of Aro-Ang3, an Investigational Rna Interference Targeting Hepatic Angiopoietin-like Protein 3, in Patients with Hypercholesterolemia. Circulation 142, A15751. 10.1161/circ.142.suppl_3.15751

[B109] Watts G. F. Schwabe C. Scott R. Gladding P. Sullivan D. R. Baker J. (2019). Rna Interference Targeting Hepatic Angiopoietin-like Protein 3 Results in Prolonged Reductions in Plasma Triglycerides and Ldl-C in Human Subjects. Circ. Baltim. 140 (25), E987–E8.

[B110] Wu N.-Q. Guo Y.-L. Zhu C.-G. Gao Y. Sun J. Xu R.-X. (2019). Poor Response to Alirocumab in a Patient with Homozygous Familial Hypercholesterolemia. Am. J. Ther. 26 (6), e743–e745. 10.1097/MJT.0000000000000898 31241493

[B111] Wu N. Q. Guo Y. L. Xu R. X. Liu J. Zhu C. G. Jiang L. X. (2013). Acute Myocardial Infarction in an 8-Year Old Male Child with Homozygous Familiar Hypercholesterolemia: Laboratory Findings and Response to Lipid-Lowering Drugs. Clin. Lab. 59 (7-8), 901–907. 10.7754/clin.lab.2012.121104 24133922

[B112] Xu C. L. Ruan M. Z. C. Mahajan V. B. Tsang S. H. (2019). Viral Delivery Systems for Crispr. Viruses 11 (1), 28. 10.3390/v11010028 PMC6356701 30621179

[B113] Xu Y. Guo J. Zhang L. Miao G. Lai P. Zhang W. (2022). Targeting Apoc3 Paradoxically Aggravates Atherosclerosis in Hamsters with Severe Refractory Hypercholesterolemia. Front. Cardiovasc. Med. 9, 840358. 10.3389/fcvm.2022.840358 35187136 PMC8847384

[B114] Yang D. Zhang W. Zhang H. Zhang F. Chen L. Ma L. (2020). Progress, Opportunity, and Perspective on Exosome Isolation - Efforts for Efficient Exosome-Based Theranostics. Theranostics 10 (8), 3684–3707. 10.7150/thno.41580 32206116 PMC7069071

[B115] Yin H. Song C.-Q. Suresh S. Wu Q. Walsh S. Rhym L. H. (2017). Structure-Guided Chemical Modification of Guide Rna Enables Potent Non-viral *In Vivo* Genome Editing. Nat. Biotechnol. 35 (12), 1179–1187. 10.1038/nbt.4005 29131148 PMC5901668

[B116] Yu Q. Zheng H. Zhang Y. (2021). Inducible Degrader of Ldlr: A Potential Novel Therapeutic Target and Emerging Treatment for Hyperlipidemia. Vasc. Pharmacol. 140, 106878. 10.1016/j.vph.2021.106878 34015522

[B117] Zhao H. Li Y. He L. Pu W. Yu W. Li Y. (2020). *In Vivo* AAV-CRISPR/Cas9-Mediated Gene Editing Ameliorates Atherosclerosis in Familial Hypercholesterolemia. Circulation 141 (1), 67–79. 10.1161/CIRCULATIONAHA.119.042476 31779484

[B118] Zheng J. Huynh H. Umikawa M. Silvany R. Zhang C. C. (2011). Angiopoietin-Like Protein 3 Supports the Activity of Hematopoietic Stem Cells in the Bone Marrow Niche. Blood 117 (2), 470–479. 10.1182/blood-2010-06-291716 20959605 PMC3031476

